# Supplementary material for: Switching magnon chirality in artificial ferrimagnet
Source: Nat Commun. 2022 Mar 10;13:1264. doi: 10.1038/s41467-022-28965-7 (PMC8913739; doi:10.1038/s41467-022-28965-7)
Supplement: Supplementary file 1 — Supplementary Information [file 41467_2022_28965_MOESM1_ESM.pdf]

Supplementary Information for:
Switching magnon chirality in artificial antiferromagnet

Yahui Liu[1], Zhengmeng Xu[1], Lin Liu[1], Kai Zhang[1], Yang Meng[2,3*], Yuanwei Sun[1,4], Peng Gao[1,4], Hong-Wu Zhao[2,3,5], Qian Niu[6*] & J. Li[1*]

[1]*International Center for Quantum Materials, School of Physics, Peking University, Beijing 100871, China*

[2]*Beijing National Laboratory for Condensed Matter Physics, Institute of Physics, Chinese Academy of Sciences, Beijing 100190, China*

[3]*School of Physical Sciences, University of Chinese Academy of Sciences, Beijing 100049, China*

[4]*Electron Microscopy Laboratory, School of Physics, Peking University, Beijing 100871, China*

[5]*Songshan Lake Materials Laboratory, Dongguan 523808, China*

[6]*Department of Physics, University of Science and Technology of China, Hefei 230026, China*

*Correspondence to: ymeng@iphy.ac.cn, niuqian@ustc.edu.cn, jiali83@pku.edu.cn

Contents

Supplementary Note 1. Cross-sectional composition maps in Al_2O_3/Gd/Py/Gd/Py/Cu sample

Supplementary Note 2. The critical field (H_{twist}) at $T > T_M$

Supplementary Note 3. The reversal polarity of anomalous Hall resistance at T_M

Supplementary Note 4. Quantitative measurements of spin pumping

Supplementary Note 5. Spin Rectification Effect and self-pumping in Py/Gd multilayer

Supplementary Note 6. Micromagnetic simulation of the static and dynamic properties of Py/Gd multilayer

Supplementary Note 7. Coexistence of right-handed and left-handed modes for $T > T_M$

Supplementary Note 8. Spin mixing conductance at Gd/Pt interface

References

Supplementary Note 1. Cross-sectional composition maps in Al_2O_3/Gd/Py/Gd/Py/Cu sample

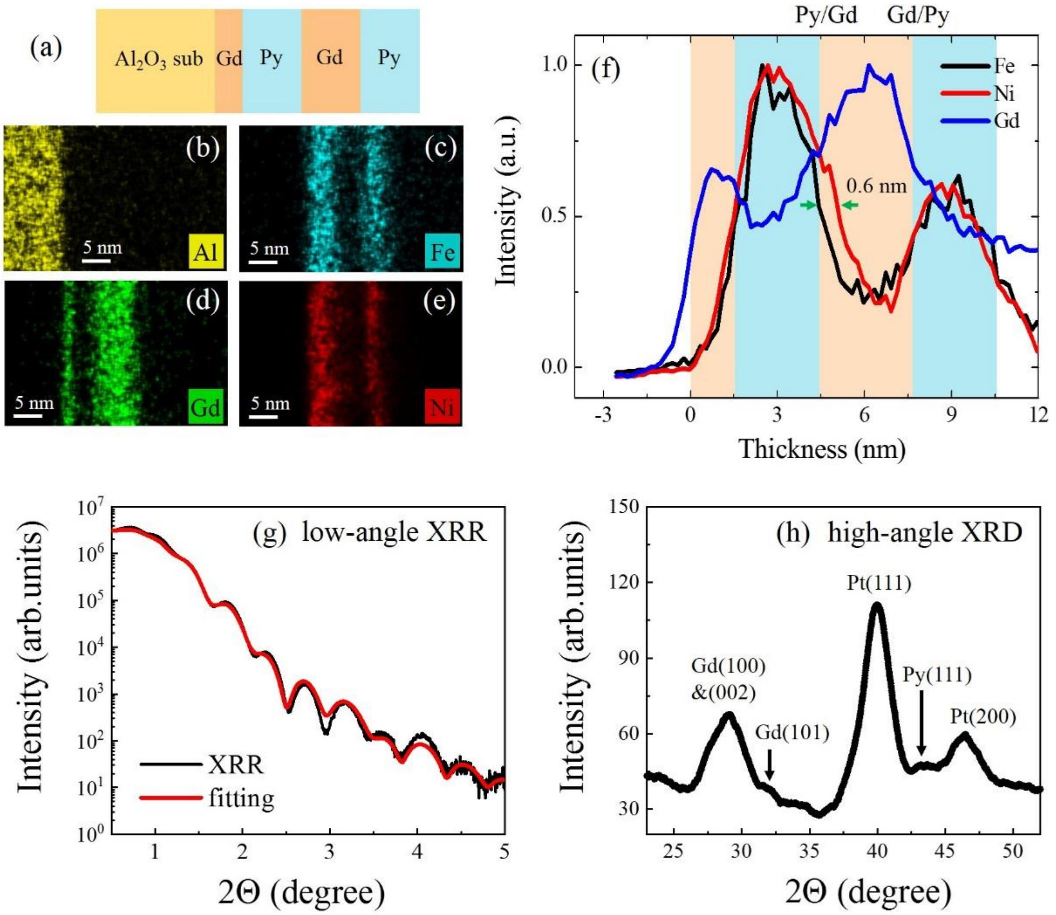

Supp. Fig. 1: Elemental distributions in Al_2O_3/Gd/Py/Gd/Py/Cu sample probed by energy dispersive X-ray spectroscopy (EDS). (a) schematic of Al_2O_3/Gd(1.5 nm)/Py(3 nm)/Gd(3 nm)/Py(3 nm)/Cu sample. EDS maps of (b) element Al, (c) element Fe, (d) element Gd and (e) element Ni, (f) element intensity profiles along the film normal direction, the interfacial intermixing between Ni and Gd is illustrated. (g) Low-angle x-ray reflectivity (XRR) scan. (h) High-angle x-ray diffraction (XRD).

The elemental distributions of Al_2O_3/Gd/Py/Gd/Py/Cu sample were probed by energy dispersive X-ray spectroscopy (EDS). Supplementary Figure 1 (b) to (e) show the EDS maps of elements Al, Fe, Gd and Ni. The EDS map of Gd illustrates two continuous and uniform Gd layers (1.5 nm and 3 nm). In addition, the Fe elemental distribution in Py layers was also uniform and a clear boundary between Fe and Gd was presented in Supp. Fig. 1 (c). The Fe intensity shows a steep drop exactly at Py/Gd interface (Supp. Fig. 1 (f)), evidencing no intermixing between Fe and Gd at Py/Gd interface. In contrast, Ni intensity drops at the inner Gd layer which is 0.6 nm away from Py/Gd interface, demonstrating the interfacial intermixing between Ni and Gd. Such interfacial intermixing is absent at Gd/Py interface. Thus the mirror symmetry of the Py/Gd/Py trilayer is broken and the Py/Gd interface and the Gd/Py interface are asymmetric. This asymmetry in Py/Gd multilayer has been reported in literatures [1,2], which might cause the difference in the magnitudes of self-pumping signals between Py/Gd and Gd/Py samples.

The quality of the sample is also characterized by the low-angle x-ray reflectivity (XRR) and the high-angle x-ray diffraction (XRD). As shown in (g), the periodical oscillations observed in the XRR scans confirm the well-defined interfaces of Py/Gd multilayer, in consistent with the result of EDS mapping. The fitting of XRR reveals a smooth Py/Gd interface with an RMS roughness of 0.49 nm. The high-angle XRD scan shows that the Py/Gd multilayer sample is polycrystalline. Different textures were observed for Gd layer and Pt capping layer.

Supplementary Note 2. The critical field (H_{twist}) at $T > T_M$

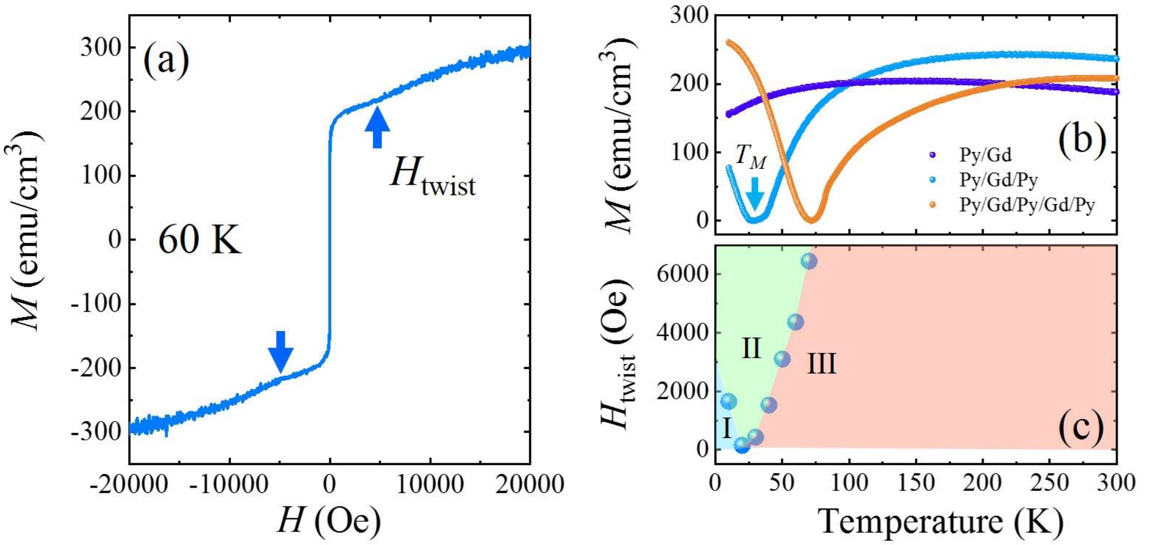

Supp. Fig. 2: (a) In-plane hysteresis loop of Py(2.5 nm)/Gd(3 nm)/Py(2.5 nm) trilayer at T = 60 K. (b) Temperature dependent in-plane magnetization of Py(2.5)/Gd(3) bilayer, Py(2.5)/Gd(3)/Py(2.5) trilayer, Py(2.5)/Gd(3)/Py(2.5)/Gd(3)/Py(2.5) sample (thickness in nm) at H = 1000 Oe, the green arrow marks the compensation temperature T_M of Py/Gd/Py trilayer. (c) Temperature evolution of H_{twist} and phase diagram of this sample. Gd-aligned phase (region I), twisted state (region II) and Py-aligned phase (region III) were identified in different colored shadows.

The in-plane magnetization of Py(2.5 nm)/Gd(3 nm)/Py(2.5 nm)/Cu(6 nm) sample was studied at different temperatures. Supp. Fig. 2 (a) shows a typical hysteresis loop at T = 60 K. Twisted state is initialized at the critical field (H_{twist}). The temperature dependence of in-plane magnetization at H = 1000 Oe reveals the compensation temperature T_M = 30 K of this sample (Supp. Fig. 2 (b)). According to the temperature evolution of H_{twist} shown in Supp. Fig. 2 (c), Gd-aligned phase (region I) and twisted state (region II) as well as Py-aligned phase (region III) can be obtained by tuning the temperature and external field H. According to Fig. 2(c) and Supp. Fig. 2 (c), Py-aligned phase is always favorable for $T \gg T_M$ unless applying an extremely large magnetic field. On the contrary, the temperature evolution of H_{twist} for $T < T_M$ is always sluggish with respect to that for $T > T_M$. Such strong asymmetry in the temperature evolution of H_{twist} for $T < T_M$ and $T > T_M$ has been reported in Fe/Gd multilayer, which is due to the energy difference in the surface twisting and the bulk twisting of Fe (or Py) moments and Gd moments [3]. H_{twist} rises less abruptly in Py/Gd/Py sample than in Py/Gd/Py/Gd/Py sample, indicating a less rigid ferrimagnetic order in Py/Gd/Py trilayer. Thus we could produce an even more rigid ferrimagnetic order by increasing the repetition number of Py/Gd multilayer. It's worth noting that the monotonic decline of H_{twist} is synchronized with the reduction of the total magnetization when approaching T_M, owing to the spin-flop transition near T_M[4].

As shown in (b), the minimum number of stacks is Py/Gd/Py trilayer to achieve the Gd-aligned phase. The Py/Gd/Py/Gd/Py multilayer shows more well-defined compensation magnetization with respect to Py/Gd/Py trilayer. And Gd-aligned phase is not accessible in Py/Gd bilayer.

Supplementary Note 3. The reversal polarity of anomalous Hall resistance at T_M

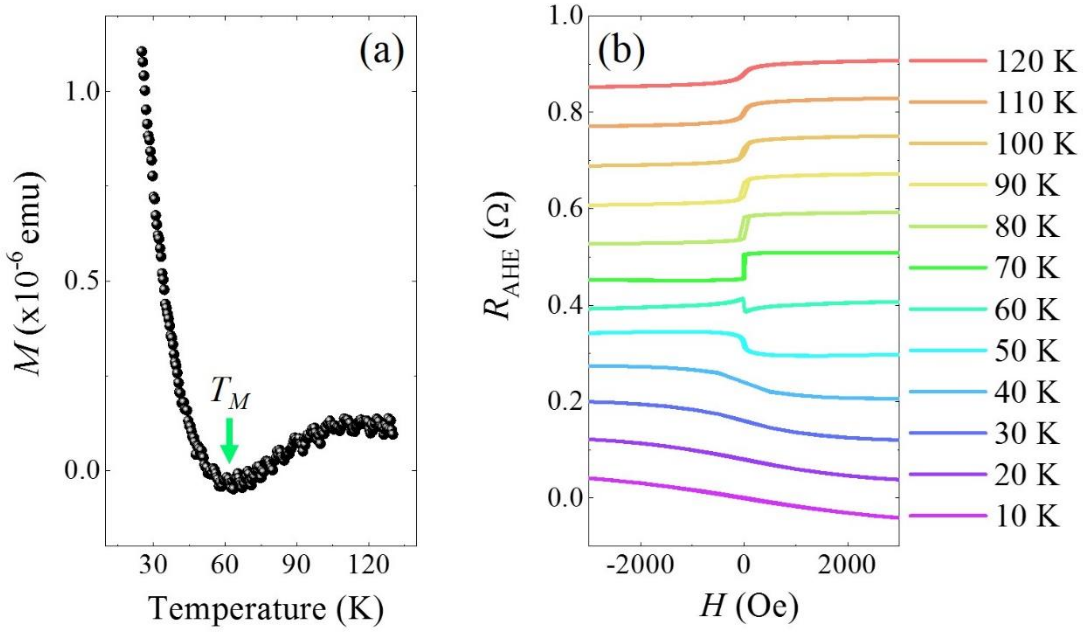

Supp. Fig. 3: (a) The temperature evolution of the in-plane magnetization of [Py(0.4)/Gd(0.8)]$_3$/Py(0.4)/Cu(6) sample (in nm) at 1000 Oe. (b) Anomalous Hall resistance R_{AHE} at a series of temperatures.

The in-plane magnetization of [Py(0.4)/Gd(0.8)]$_3$/Py(0.4)/Cu(6) sample (in nm) was measured at 1000 Oe in the temperature range between 20 and 150 K. The compensation temperature T_M = 60 K was depicted in Supp. Fig. 3 (a). The Hall resistance of this sample was measured as a function of the out-of-plane (OOP) magnetic field. After the linear fitting of ordinary Hall effect (OHE) at high magnetic field (between 8 T and 9 T), we subtracted OHE from the signals and obtained the Hall resistance solely caused by anomalous Hall effect (AHE) R_{AHE}. It has been widely accepted that AHE of rare-earth-transition-metal ferrimagnet is governed by the transition metal [5]. R_{AHE} is proportional to the OOP component of the transition-metal magnetization. The 4f shell, which dominates the magnetic properties of rare-earth metal, is located far below the Fermi level [6].

As shown in Supp. Fig. 3 (b), the polarity of R_{AHE} is reversed exactly at T_M = 60 K, unambiguously confirming the reversal of Py magnetization with respect to the external magnetic field across T_M. The compensation temperature T_M concretely manifests the transition between Py-aligned phase and Gd-aligned phase. R_{AHE} is not fully saturated in Supp. Fig. 3 (b) because the easy magnetization direction of this sample is in the sample plane. Py magnetic moment is oblique to the normal direction during the R_{AHE} measurements.

Supplementary Note 4. Quantitative measurements of spin pumping

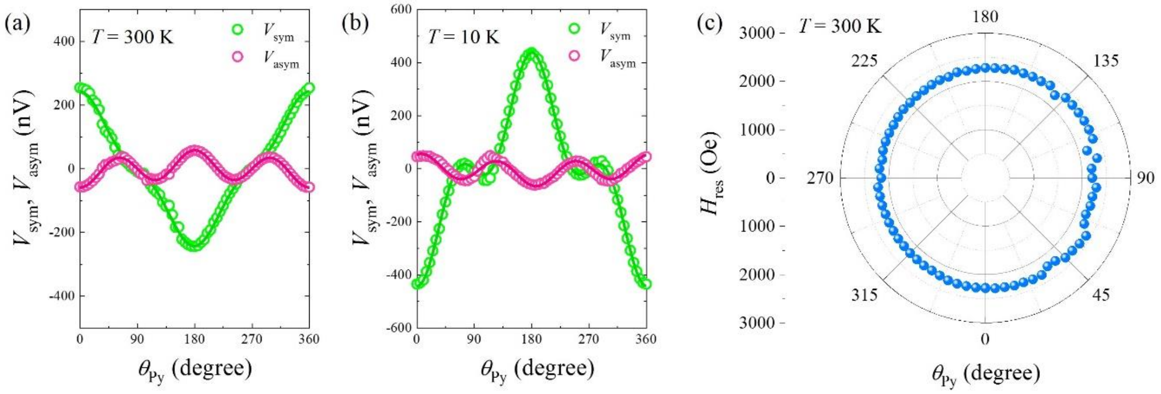

Supp. Fig. 4: Angular dependence of V_{sym} and V_{asym} (a) at T = 300 K and (b) at T = 10 K. The quantitative fittings were carried out by taking SRE into consideration. (c) Angular dependence of H_{res} at T = 300 K. The resonance field H_{res} is isotropic against θ_{Py}. The microwave frequency f is fixed at 13 GHz in these measurements.

The pure spin current across Py/Pt interface of the Py/Gd multilayer sample was generated and studied through spin pumping, recording the voltage signals $V(H)$ by sweeping in-plane magnetic field (H). In order to distinguish the spin pumping voltage signals V_{sp} from Spin Rectification Effect (SRE), such as anisotropic magnetoresistance (AMR) and anomalous Hall effect (AHE), we carried out angular dependent measurements of $V(H)$ signals following the conventional method in literatures [7,8].

$V(H)$ signals are fitted by the combination of symmetric Lorentzian curve and antisymmetric Lorentzian curve.

$$V(H) = V_{sym}\frac{(\Delta H)^2}{(H-H_{res})^2+(\Delta H)^2} + V_{asym}\frac{-2\Delta H(H-H_{res})}{(H-H_{res})^2+(\Delta H)^2} \quad (1)$$

Where V_{sym} and V_{asym} are the amplitude of symmetric curve and antisymmetric curve, respectively. H_{res} is the resonance field and ΔH is the linewidth corresponding to a half-width at half-maximum (HWHM). Figure 3(d) and (e) show the best fitting of $V(H)$ signals at T = 300 K and T = 10 K, respectively. ΔH is ~ 150 Oe at 300 K and ~ 500 Oe at 10 K (f = 13 GHz), which is comparable with the literatures' results. A broader linewidth ΔH is observed at T = 10 K with respect to that at T = 300 K, due to the enhanced magnetic damping at low temperature. Such enhancement of magnetic damping originates from the significant magnetostriction of Gd film, while Gd has null magnetocrystalline anisotropy because of its null orbital moment [9,10]. We can also extract the Gilbert damping parameter which is 0.012 for Cu capping sample where spin pumping doesn't occur at Py/Cu interface. This value is in excellent agreement with the values reported in literatures. Angular dependences of V_{sym} and V_{asym} at T = 300 K and T = 10 K are plotted in Supp. Fig. 4(a) and (b). To extract the V_{sp} signals quantitatively, SRE induced by AMR and AHE are taken into account during the fitting.

$$V_{sym} = V_{sp}cos^3(\theta_M) + V_{AHE}^{sym}cos(\theta_M) + V_{AMR}^{sym}cos(2\theta_M)cos(\theta_M) \quad (2)$$

$$V_{asym} = V_{AHE}^{asym}cos(\theta_M) + V_{AMR}^{asym}cos(2\theta_M)cos(\theta_M) \quad (3)$$

Where V_{sp} is the spin pumping signals due to the pure spin current. V_{AHE}^{sym} and V_{AMR}^{sym} are the symmetric components induced by AHE and AMR, respectively. V_{AHE}^{asym} and V_{AMR}^{asym} are the

antisymmetric components induced by AHE and AMR, respectively. θ_M is the azimuthal angle of magnetization. The Py/Gd multilayer is a polycrystalline film sample, magnetic anisotropy is expected to be absent in this system. This statement is supported by the isotropic angular dependence of $H_{\rm res}$ at T = 300 K (Supp. Fig. 4(c)). Thus θ_M is equivalent to the azimuthal angle of external field $\theta_{\rm H}$ in our measurements. Specifically, $\theta_{\rm Py}$ equals $\theta_{\rm H}$ at T = 300 K and $\theta_{\rm H}$ - 180° at T = 10 K. For T = 300 K, the best fitting of $V_{\rm sym}$ yields $V_{\rm sp}$ = 282 nV, V_{AMR}^{sym} = -71 nV, V_{AHE}^{sym} = 31 nV, and V_{AMR}^{asym} = -85 nV, V_{AHE}^{asym} = 26 nV of $V_{\rm asym}$ fitting. The fitting results for T = 10 K are the following: $V_{\rm sp}$ = -371 nV, V_{AMR}^{sym} = -91 nV, V_{AHE}^{sym} = 23 nV, and V_{AMR}^{asym} = 85 nV, V_{AHE}^{asym} = -24 nV. It's worth mentioning that $V_{\rm sp}$ signal and SRE signal add up destructively at T = 300 K and constructively at T = 10 K, therefore $V_{\rm sp}$ is slightly higher than $V_{\rm sym}$ at T = 300 K and lower than $V_{\rm sym}$ at T = 10 K.

In light of Py-aligned phase at T = 300 K and Gd-aligned phase at T = 10 K, the $V_{\rm sp}$ signals against $\theta_{\rm Py}$ have the opposite polarities in spite of the same $M_{\rm Py}$ orientations. Thus we can conclude that the $V_{\rm sp}$ polarity of spin pumping is determined by the chirality of spin precession rather than the spin orientation. In addition, the microwave power $P_{\rm app}$ applied on the sample is $P_{\rm app}$ = 3.5 mW for T = 300 K and $P_{\rm app}$ = 4.5 mW for T = 10 K. Thus the spin pumping efficiency $V_{\rm sp}/P_{\rm app}$ are comparable at these two temperatures. It's worth mentioning that the phases of SRE signals are opposite for T = 300 K and T = 10 K, as the result of the phase shifting between the precessional component of $M_{\rm Py}$ and the induction current. To our surprise, the phase of induction microwave current can be modified by varying the temperature, which requires the further investigation in future.

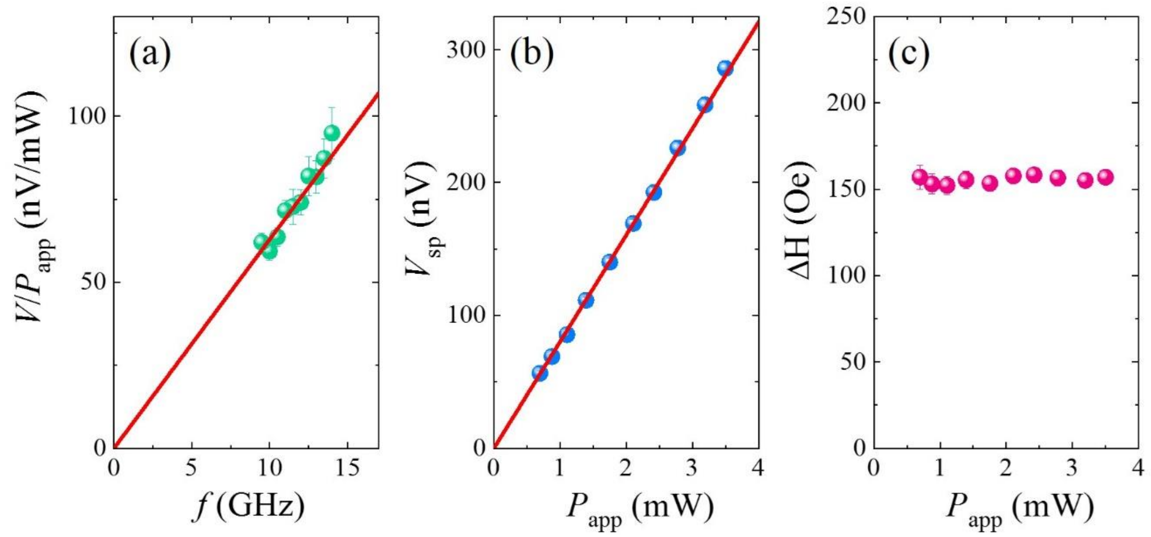

Supp. Fig. 5: (a) The normalized $V_{\rm sp}$ signal ($V/P_{\rm app}$) is linearly proportional to f. (b) $V_{\rm sp}$ signals against the microwave power $P_{\rm app}$ applied on the sample at T = 300 K. (c) Linewidth ΔH is barely changed versus $P_{\rm app}$.

Supplementary Figure 5(b) plots the $V_{\rm sp}$ signals against the microwave power $P_{\rm app}$ applied on the sample at T = 300 K. The linear dependence presented here rules out the possible nonlinear damping in our experiments [11]. This statement is further supported by Supp. Fig. 5(c) that the linewidth ΔH is barely changed versus $P_{\rm app}$. The $V_{\rm sp}$ signals are generated as the result of the uniform $M_{\rm Py}$ precession in the outermost Py layer.

Supplementary Note 5. Spin Rectification Effect and self-pumping in Py/Gd multilayer

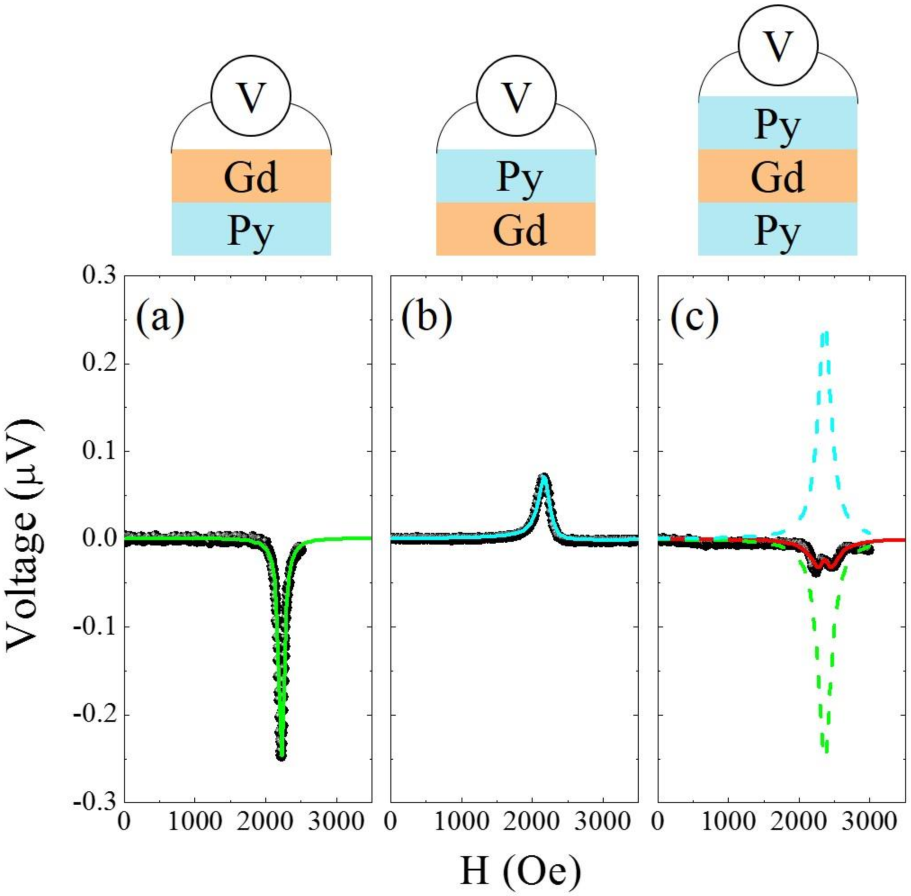

Supp. Fig. 6: Spin pumping *V*(*H*) data of (a) Al_2O_3/Py/Gd/Cu, (b) Al_2O_3/Cu/Gd/Py/Cu and (c) Al_2O_3/Py/Gd/Py/Cu samples at 13 GHz. The line shape of *V*(*H*) data was fitted by symmetric Lorentzian curve for Al_2O_3/Py/Gd/Cu (green peak in (a)) and Al_2O_3/Cu/Gd/Py/Cu (cyan peak in (b)). The red fitting curve in (c) exhibits the superposition of two *V*(*H*) signals with opposite sign from Py/Gd and Gd/Py bilayers. The compensated self-pumping was observed in Al_2O_3/Py/Gd/Py/Cu.

The voltage signals *V*(*H*) were mainly attributed to the spin pumping at Py/Pt interface and inverse spin Hall effect (ISHE) of Pt layer. However, it has been well studied that spin pumping signals can be contaminated by Spin Rectification Effect (SRE) [7], including anisotropic magnetoresistance (AMR) and anomalous Hall effect (AHE) [8,12]. In addition, ferromagnetic metal alone may produce a voltage signal via self-pumping [13]. To evaluate and exclude the possible contributions of SRE or self-pumping, we measured *V*(*H*) of several control samples without Pt electrode at room temperature. Supplementary Figure 6 (a) shows the voltage signal *V*(*H*) of Al_2O_3/Py/Gd/Cu sample. The negative *V*(*H*) was observed when sweeping in-plane magnetic field, which may result from SRE or self-pumping in Py/Gd bilayer sample. On the contrary, the positive *V*(*H*) of a smaller magnitude was observed in Al_2O_3/Cu/Gd/Py/Cu sample (Supp. Fig. 6 (b)). According to the opposite sign of *V*(*H*) shown in Supp. Fig. 6 (a) and (b), these *V*(*H*) signals should be dominated by the self-pumping in Py/Gd and Gd/Py bilayers. SRE signals should maintain the same sign of *V*(*H*) due to the unchanged sign of the inductive rf current, in conjunction with the two-fold symmetry of AMR and AHE during the inversion of Py layer and Gd

layer. Different $V(H)$ magnitudes of Py/Gd and Gd/Py might result from the different qualities of Py/Gd and Gd/Py interfaces due to the different growth procedures. A larger $V(H)$ magnitude is expected if a better Gd/Py interface is obtained. SRE signals are inevitable and might also contribute to the different $V(H)$ magnitudes of Py/Gd and Gd/Py. The contributions of Cu capping layers were omitted. Supplementary Figure 6 (c) shows $V(H)$ of Al_2O_3/Py/Gd/Py/Cu sample where two tiny negative peaks were observed around the resonance field. These two negative peaks stem from the superposition of two self-pumping signals from Py/Gd (green dashed peak) and Gd/Py (cyan dashed peak). In another word, self-pumping signals of Py/Gd and Gd/Py compensate each other and yield a negligible voltage signal (33 nV) with respect to V_{sp} of Py/Pt. This uncompensated voltage signal (33 nV) established the upper limit of SRE voltage signal in this Al_2O_3/Py/Gd/Py/Cu sample.

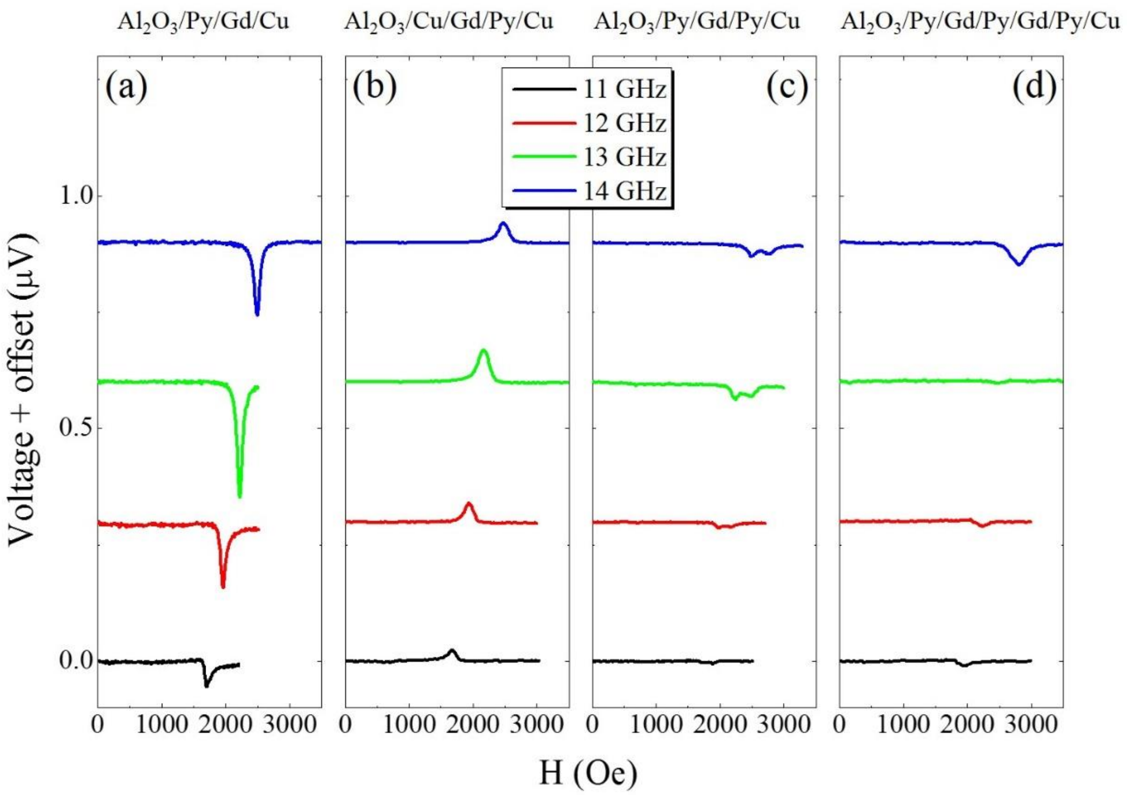

Supp. Fig. 7: Spin pumping $V(H)$ data of (a) Al_2O_3/Py/Gd/Cu, (b) Al_2O_3/Cu/Gd/Py/Cu, (c) Al_2O_3/Py/Gd/Py/Cu and (d) Al_2O_3/Py/Gd/Py/Gd/Py/Cu samples at various microwave frequencies (11 GHz, 12 GHz, 13 GHz and 14 GHz). $V(H)$ data were shifted vertically by an offset for a better visibility of data.

To further demonstrate the compensated self-pumping of Py/Gd multilayer, we measured $V(H)$ of several control samples at different microwave frequencies (11 GHz, 12 GHz, 13 GHz and 14 GHz). The opposite sign of $V(H)$ in Py/Gd and Gd/Py bilayers was further confirmed, which was independent of the microwave frequencies. The tiny negative double-peak was observed in Al_2O_3/Py/Gd/Py/Cu sample, and such negative double-peak became further small and negligible in Al_2O_3/Py/Gd/Py/Gd/Py/Cu sample except for that at 14 GHz. The visible $V(H)$ signal with the magnitude of 45 nV at 14 GHz established the upper limit of SRE voltage signal in this sample (Supp. Fig. 7 (d)). In consequence, the spin pumping voltage signals V_{sp} in Al_2O_3/Py/Gd/Py/Gd/Py/Pt sample were mainly attributed to the spin pumping at Py/Pt interface

and inverse spin Hall effect (ISHE) of Pt layer. The compensated self-pumping was achieved in Py/Gd multilayers without Pt electrode.

Our Py/Gd multilayer samples were in Py-aligned phase at room temperature while M_{Py} was much larger than M_{Gd}. Thus spin pumping from Py into Gd is expected to dominate self-pumping of Py/Gd multilayers. However, in light of the non-zero spin Hall angle of Py, the spin current backflow from Gd into Py should be taken into consideration as well [14]. The further investigation is required for the deep understanding of self-pumping in Py/Gd multilayers, which is beyond the scope of this work.

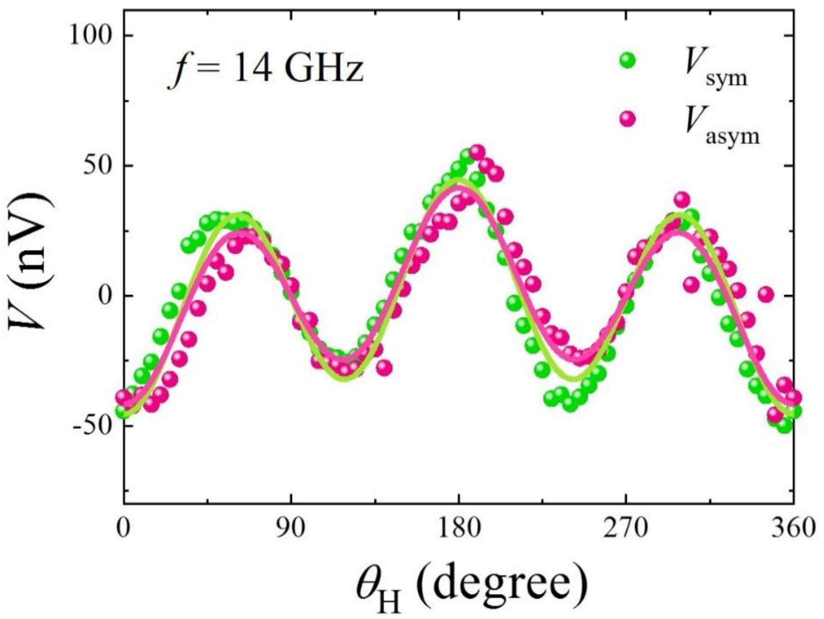

Supp. Fig. 8: Angular dependence of V_{sym} and V_{asym} for Al_2O_3/Py/Gd/Py/Gd/Py/Cu sample at f = 14 GHz.

The visible $V(H)$ signal is ~ 45 nV at 14 GHz for Al_2O_3/Py/Gd/Py/Gd/Py/Cu sample (Supp. Fig. 7 (d)). The angular dependence of this $V(H)$ signal is presented in Supp. Fig. 8. The V_{sym} and V_{asym} signals have the same phase and similar amplitudes. The quantitative fitting yields the following results V_{AMR}^{sym} = -72 nV, V_{AHE}^{sym} = 27 nV and V_{AMR}^{asym} = -60 nV, V_{AHE}^{asym} = 18 nV, in accordance with the SRE signals obtained for Al_2O_3/Py/Gd/Py/Gd/Py/Pt sample (Supp. Fig. 4 (a)). According to these observations, the capping layer (Pt or Cu) has little impact on the SRE signals of Py/Gd multilayer, the SRE in this system is a bulk effect and dominated by the ferromagnetic multilayer. The SRE $V(H)$ signal has the critical dependence on the microwave frequency (visible at 14 GHz and negligible at 13 GHz), which is an interesting topic and requires the further investigation in future.

To manifest SRE signals for the scenario where both FMR mode and exchange mode exist, we measured the $V(H)$ signals of the Py/Gd multilayer (T = 20 K and f = 14 GHz) with Pt capping layer and Cu capping layer, respectively (Supp. Fig. 9 (a) and (b)). The V_{sp} signals govern the $V(H)$ signals of the Pt capping sample, while the $V(H)$ signals of the Cu capping sample are fully determined by SRE. The $V(H)$ signals are normalized by the microwave power P_{app} applied on the samples. According to the quantitative fitting of $V(H)$ signals at T = 10 K, the $V(H)$ signals of FMR mode are mainly attributed to spin pumping signals V_{sp}, which is also true for other temperatures.

As shown in Supp. Fig. 9 (a) and (b), the negative $V(H)$ signals of FMR mode are observed at $H \sim$ 2000 Oe for both samples at T = 20 K, where the $V(H)$ signal of Pt capping sample is mainly assigned to V_{sp} and the $V(H)$ signal of Cu capping sample is due to SRE. The V_{sp} signal and SRE signal are both negative and add up constructively, in agreement with the result at T = 10 K shown in Fig. 3(e). Thus the real V_{sp} signal is slightly smaller than $V(H)$ signal at T = 10 K and T = 20 K. On the contrary, the $V(H)$ signals at $H \sim$ 6500 Oe of exchange mode have completely different symmetries for Pt capping sample and Cu capping sample. The $V(H)$ signal of Pt sample is dominated by V_{sym} component, accompanied by a minor V_{asym} component of ~ -20 nV/mW. The $V(H)$ signal of Cu sample is perfectly described by a V_{asym} component of ~ -15 nV/mW. Accordingly, SRE cause a V_{asym} component in $V(H)$ signal of Pt sample and make no influence on V_{sym} component. The major $V(H)$ signal of exchange mode at T = 20 K is again attributed to the V_{sp} signal. To our surprise, the symmetries of SRE signals of exchange mode change when varying the frequency (Supp. Fig. 9(c)). The SRE signals are dominated by V_{asym} component at 12 GHz and 14 GHz, and by V_{sym} component at 16 GHz. Meanwhile, the magnitudes of SRE signals are ~ 10 nV/mW at 12 GHz, ~ 25 nV/mW at 14 GHz and ~ 13 nV/mW at 16 GHz, all of which are one order of magnitude smaller than the $V(H)$ signals of Pt sample (Fig. 4(a)) and depend nonmonotonically on frequency. In analogy with the $V(H)$ signals at T = 20 K, the SRE signals at T = 30 K cause a V_{asym} component of $V(H)$ signals at 12 GHz and 14 GHz, and a minor V_{sym} component at 16 GHz. Consequently, we can reach a qualitative conclusion that the $V(H)$ signals of exchange mode in Pt capping sample are mainly attributed to the pure spin current due to spin pumping, SRE signals make the minor contributions to $V(H)$ signals. It's worth mentioning that the quantitative evaluation of the V_{sp} signal of exchange mode is very challenging due to the coexistence of FMR mode and exchange mode. The superimposed $V(H)$ lineshapes of FMR mode and exchange mode make the quantitative fitting impracticable. The magnetic system with the well separated FMR mode and exchange mode is desirable to reach such quantitative fitting.

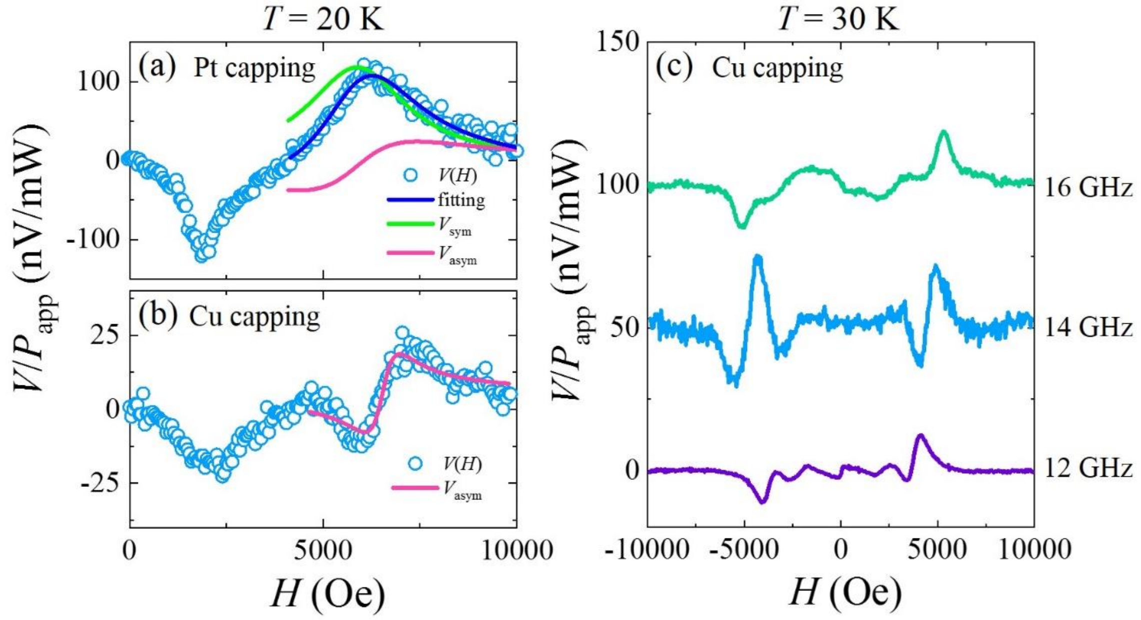

Supp. Fig. 9: The normalized $V(H)$ signals of (a) Al_2O_3/Py/Gd/Py/Gd/Py/Pt sample and (b) Al_2O_3/Py/Gd/Py/Gd/Py/Cu sample at T = 20 K and f = 14 GHz, with the best fitting of V_{sym} and V_{asym}. (c) The normalized $V(H)$ signals of Al_2O_3/Py/Gd/Py/Gd/Py/Cu sample at T = 30 K.

Supplementary Note 6. Micromagnetic simulation of the static and dynamic properties of Py/Gd multilayer

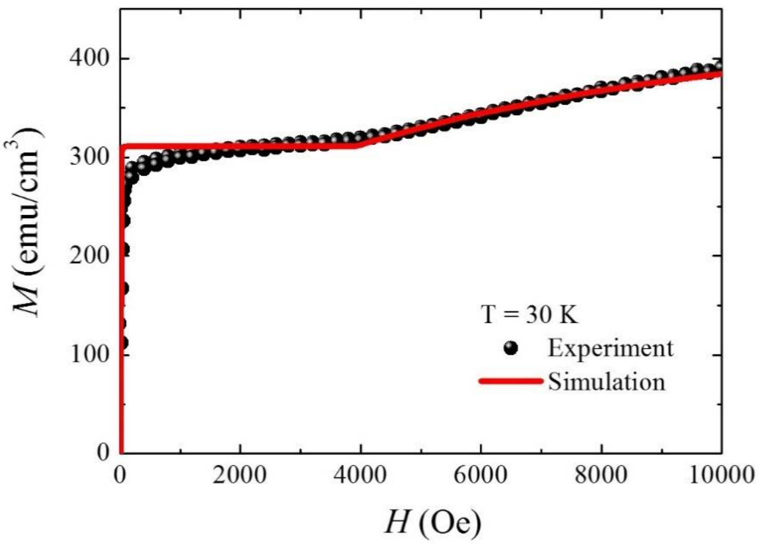

Supp. Fig. 10: The positive half branch of the hysteresis loop at 30 K obtained in experiment and in simulation accordingly, for Al_2O_3/Py(2.5)/Gd(3)/Py(2.5)/Gd(3)/Py(2.5)/Pt(6) sample (in nm).

We reproduced the positive half branch of the hysteresis loop of the Py/Gd multilayer at T = 30 K by the micromagnetic simulation using the Object Oriented MicroMagnetic Framework (OOMMF) code based on the Landau-Lifshitz-Gilbert equation [15]. The magnetic structure of Py/Gd multilayer in the twisted state is a one-dimensional spin chain winding along the film normal direction. To imitate such one-dimensional spin chain, the cell size was chosen to be 100 μm×100 μm×0.25 nm. Within each 100 μm×100 μm plane, only one spin was taken into consideration. Thus the whole system behaves like a one-dimensional spin chain. Using the micromagnetic parameters as the following, the Py/Gd multilayer sample in the simulations was established. Exchange stiffness in Py sublayer is $A_{Py} = 1\times10^{-6}$ erg/cm and M_{Py} = 810 emu/cm^3; M_{Gd} = 1750 emu/cm^3 and $A_{Gd} = 0.7\times10^{-7}$ erg/cm in Gd sublayer; the interfacial exchange stiffness is $A_{int} = -1.5\times10^{-7}$ erg/cm for the antiferromagnetic coupling at the Py/Gd interface [16]. As shown in Supp. Fig. 10, the positive half branch of the hysteresis loop was well reproduced in the simulation. The critical field (H_{twist}) to initialize the twisted state is dominated by A_{Gd} and A_{int}, the twisted spin texture forms predominately in Gd sublayer.

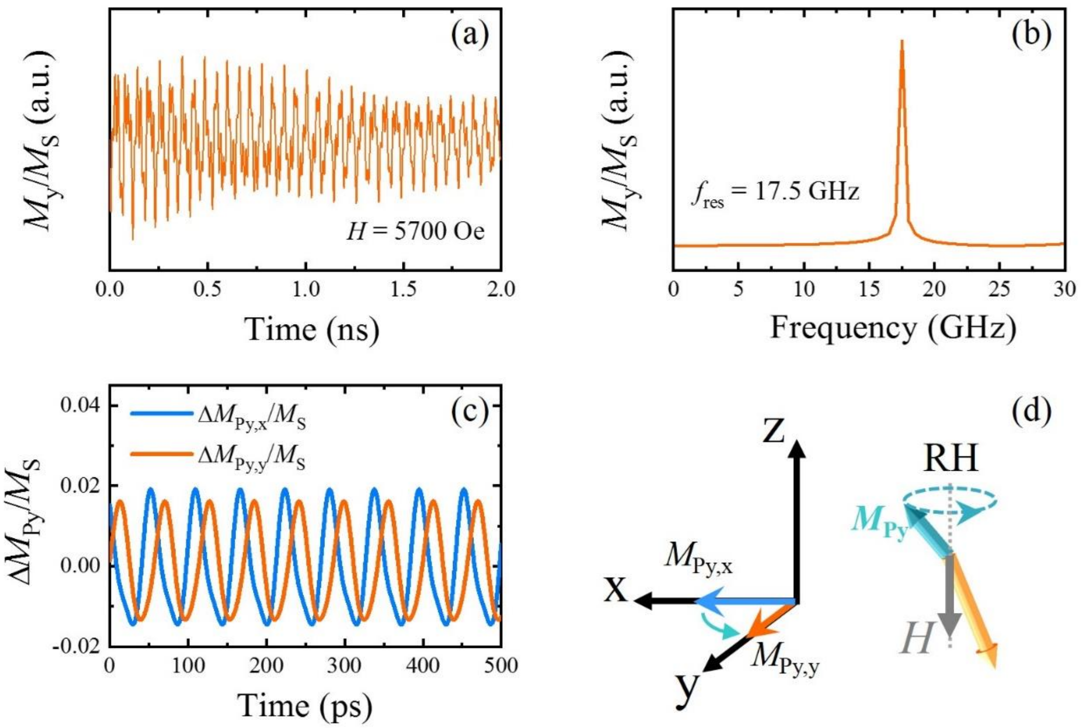

Supp. Fig. 11: (a) The oscillation of the magnetization component recorded after a pulsed magnetic field. (b) The Fast Fourier Transform of the oscillation in (a). (c) The magnetization precession in Py sublayer under a periodic sinusoidal magnetic microwave field. (d) Schematic of right-handed precession of Py magnetization in Py sublayer at f = 17.5 GHz and H = 5700 Oe.

The frequency of magnetic resonance in the Py/Gd multilayer was obtained by the ringdown method [17]. A pulsed magnetic field (10 ps) with the strength of 100 Oe along x direction was applied at the beginning of simulations. A static magnetic field of 5700 Oe was applied along -z direction to ensure the twisted state during the simulation. The in-plane and out-of-plane magnetic dynamics were recorded after this perturbation. As shown in Supp. Fig. 11 (a), the oscillation of the out-of-plane magnetization component (y-direction) is composed of the different magnetization precessions with different periods (i.e. different resonance frequencies). The salient period of this oscillation is about 50 ps while the minor shorter one could be less than 10 ps. After performing the Fast Fourier Transform (FFT) on this oscillation, the resonance frequencies of the Py/Gd multilayer were extracted (Supp. Fig. 11 (b)). The lowest resonance frequency is 17.5 GHz at H = 5700 Oe, corresponding to the exchange mode in twisted state of Py/Gd multilayer. The other resonance modes are well above 30 GHz which are beyond the scope of this work. The resonance frequency of this exchange mode depends on the strength of H_{twist}. The H dependent resonance frequency of exchange mode is revealed according to the simulations at a series of static magnetic field above H_{twist}. The simulation results are depicted in Fig. 4(b), in good agreement with the experiment data.

Then we applied a periodic sinusoidal magnetic microwave field (magnitude of 10 Oe along x-direction) of 17.5 GHz at H = 5700 Oe [18]. Supp. Fig. 11 (c) exhibits the steady magnetization precession in Py sublayer after the transient dynamics has been damped. The in-plane Py magnetic component reaches the positive maximum along x-direction in advance of the out-of-plane Py component by 90 ° during the oscillation, unambiguously demonstrating the right-handed precession of Py magnetization in twisted state (Supp. Fig. 11 (d)).

Supplementary Note 7. Coexistence of right-handed and left-handed modes for $T > T_M$

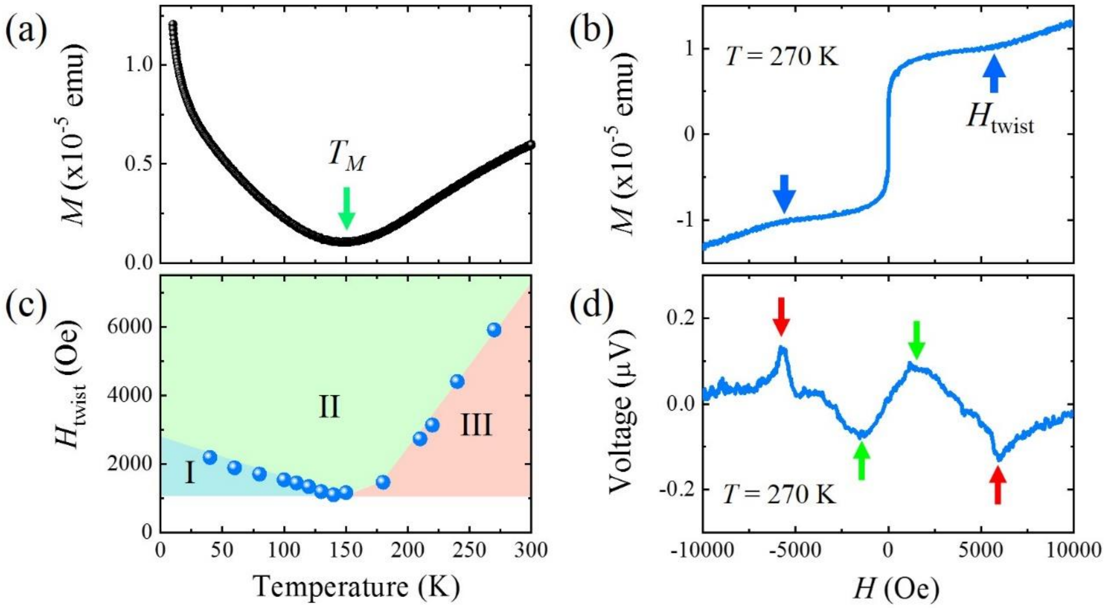

Supp. Fig. 12: (a) The temperature dependence of in-plane magnetization of MgO/Fe(2.4)/Gd(3)/Fe(2.4)/Gd(3)/Fe(2.4)/Pd(6) sample (in nm) at 1000 Oe, the compensation temperature is T_M = 150 K of this sample. (b) The hysteresis loop at T = 270 K with H_{twist} = 5900 Oe. (c) Critical field H_{twist} at a series of temperatures. Gd-aligned phase (region I), twisted state (region II) and Py-aligned phase (region III) were identified in different colored shadows. (d) The spin pumping voltage (8 GHz) at T = 270 K.

Both Gd-aligned phase and twisted state are accessible for $T < 60$ K in Py/Gd multilayer, so that we can observe the spin pumping signals of both ferromagnetic mode (Gd-aligned phase) and exchange mode (twisted state). In principle, these two modes are also expected for $T > 60$ K. Nevertheless, only ferromagnetic mode was observed in Py-aligned phase (Fig. 3) due to the extremely large H_{twist} (probably exceeding 20000 Oe) for $T \gg T_M$. To demonstrate these two modes for $T \gg T_M$ case, Fe/Gd multilayer was utilized because a much weaker H_{twist} was observed in this system [3]. Supplementary Figure 12 (a) shows the temperature dependence of in-plane magnetization of MgO/Fe(2.4)/Gd(3)/Fe(2.4)/Gd(3)/Fe(2.4)/Pd(6) sample (in nm) at 1000 Oe. A local minimum confirms T_M = 150 K of this sample. The hysteresis loop at T = 270 K clearly exhibits a nonlinear rise of the magnetization with external field, indicating the initiation of the twisted state at H_{twist} = 5900 Oe. Supplementary Figure 12 (c) summarized the strength of H_{twist} at a series of temperatures. The temperature evolutions of H_{twist} are not symmetric for $T < T_M$ and $T > T_M$, in line with that of Py/Gd multilayer except that H_{twist} is accessible for $T > T_M$ in Fe/Gd multilayer. The spin pumping voltage was measured (8 GHz) at T = 270 K. Analogous to the observations in Py/Gd multilayer for $T < T_M$, the spin pumping signals of both ferromagnetic mode (Fe-aligned phase) and exchange mode (twisted state) were observed at T = 270 K. For $H > 0$ Oe, the positive voltage signal at the lower resonance field (marked by the green arrow) corresponds to the spin pumping of ferromagnetic mode with right-handed precession of Fe magnetic moment. The negative voltage signal at the higher resonance field (marked by the red arrow) corresponds to the spin pumping of exchange mode with left-handed precession of Fe moment. The handedness of Fe moment precession determines the sign of spin pumping voltage, in agreement with the observations in Py/Gd multilayer for $T < T_M$.

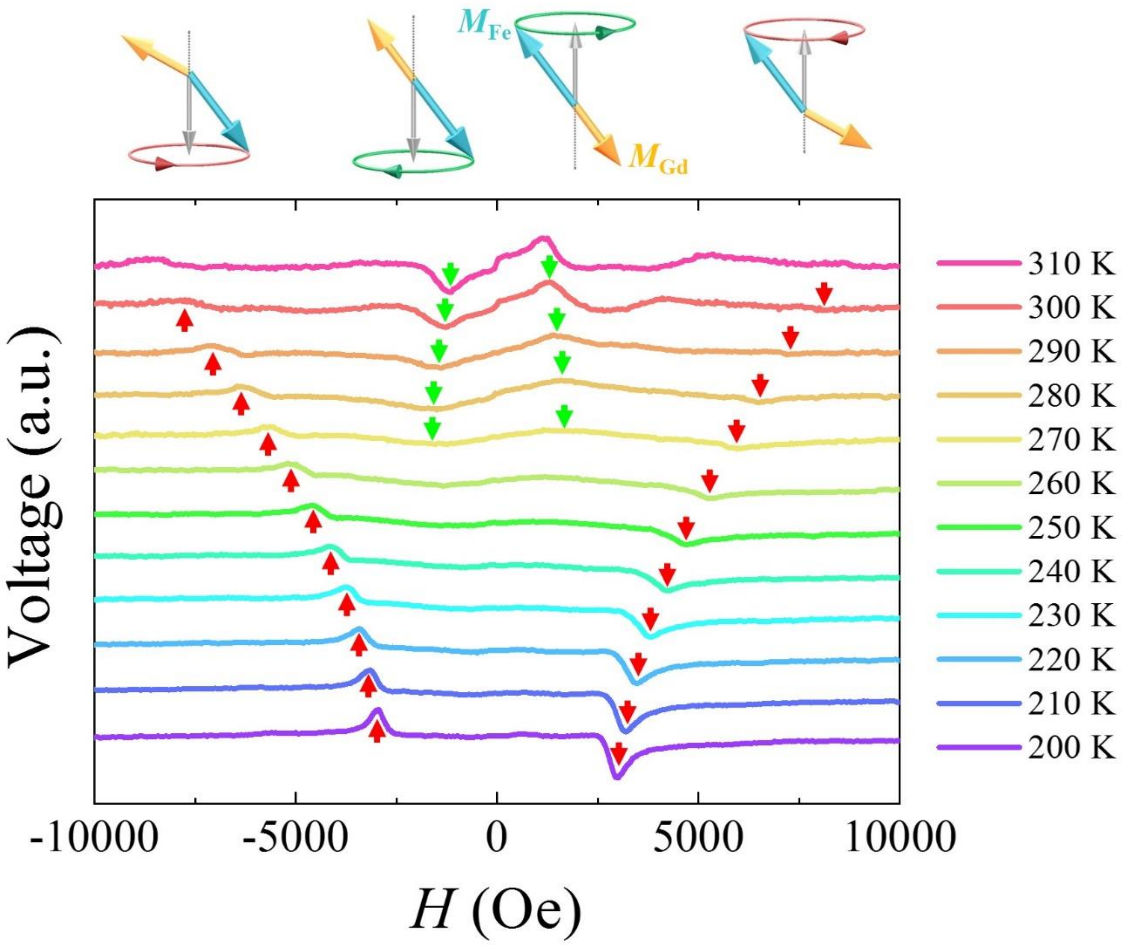

Supp. Fig. 13: The spin pumping voltage (8 GHz) at a series of temperatures above T_M.

The twisted state is unable to be achieved for $H < H_{\text{twist}}$, thus the emergence of exchange mode should depend on the strength of H_{twist} which is temperature dependent (Supp. Fig. 12 (c)). We measured the spin pumping voltages of Fe/Gd multilayer at a series of temperatures (Supp. Fig. 13). The resonance field of exchange mode at 8 GHz declines monotonically when cooling the sample, which is compatible with the temperature evolution of H_{twist}. Therefore, we can conclude that the critical field H_{twist} determines the resonance field of exchange mode and the negative voltage signals at the higher resonance field (marked by the red arrow) result from the spin pumping of left-handed precession of Fe moment in exchange mode.

Supplementary Note 8. Spin mixing conductance at Gd/Pt interface

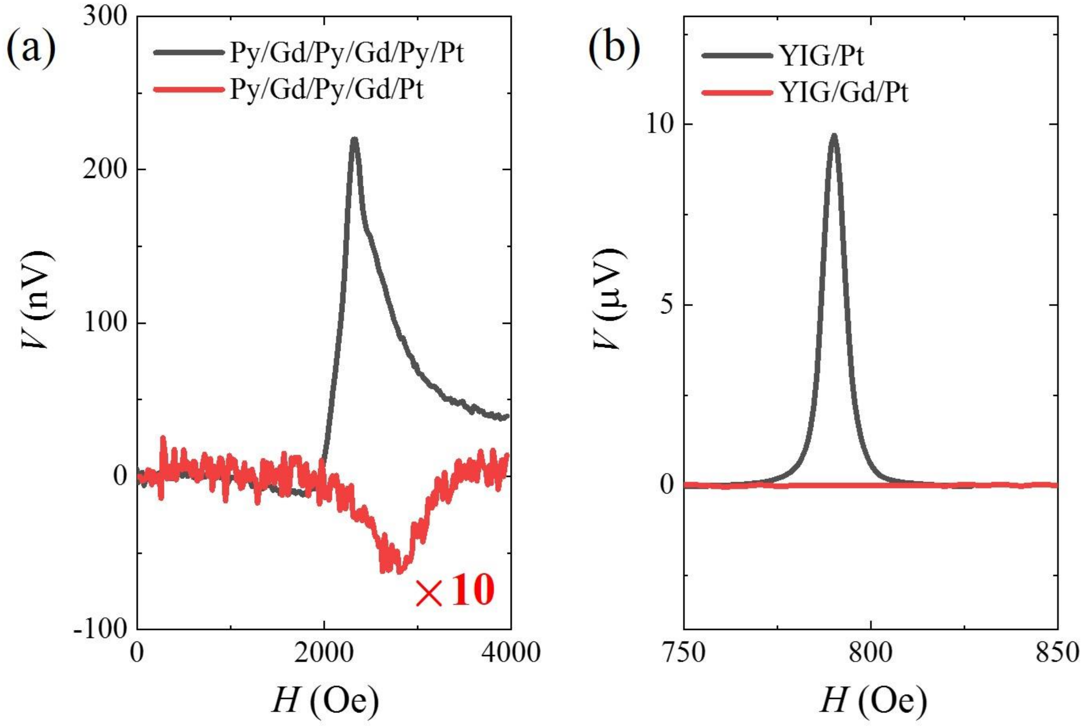

Supp. Fig. 14: The spin pumping voltages of (a) Py/Gd/Py/Gd/Py/Pt and Py/Gd/Py/Gd/Pt samples (f = 13 GHz), and (b) YIG/Pt and YIG/Gd/Pt samples (f = 4 GHz). The $V(H)$ signal of Py/Gd/Py/Gd/Pt sample is magnified by 10 for its visibility.

To rule out any possible contributions of the inner Gd and Py layers to spin pumping voltage V_{sp}, we did the comparative experiments on Py/Gd multilayer samples with Py/Pt interface and Gd/Pt interface. Similar to the result shown in Fig. 3(d), we observed a pronounced positive $V(H)$ signal for Py/Gd/Py/Gd/Py/Pt sample with Py/Pt interface. On the contrary, a tiny negative $V(H)$ signal was observed for Py/Gd/Py/Gd/Pt sample with Gd/Pt interface. As elucidated in S6 above, this negative $V(H)$ signal might rise from the self-pumping or SRE signals of Py/Gd multilayer. Taking the fact that positive $V(H)$ signal is absent for the Py/Gd multilayer sample with Gd/Pt interface, the spin mixing conductance of Gd/Pt interface is expected to be negligible.

To remove the contamination of SRE signal and strengthen the soundness of our conclusion, we replaced metallic Py layer by insulating YIG film and detected $V(H)$ signal from YIG/Pt and YIG/Gd/Pt samples. The SRE signal is expected to be minimized because YIG film is free of charge current. Indeed, the $V(H)$ signal recorded on YIG/Pt is close to a symmetric Lorentzian with respect to the $V(H)$ signal of Py/Gd multilayer. Moreover, detectable $V(H)$ signal is absent for YIG/Gd/Pt sample, confirming the negligible spin mixing conductance of Gd/Pt interface. This result also indicates that the tiny negative $V(H)$ signal of Py/Gd/Py/Gd/Pt sample rises from SRE.

In conclusion, the inner Gd and Py layers are physically separated from the Pt layer and make null contributions to spin pumping at the Py/Pt interface; only the M_{Py} precession of the outermost Py layer contributes to the $V(H)$ signals of the Pt layer.

References

1. Ranchal, R., Aroca, C., Sánchez, M., Sánchez, P. & López, E. Improvement of the structural and magnetic properties of Permalloy/Gadolinium multilayers with Mo spacers. *Appl. Phys. A* **82**, 697 (2006). DOI: 10.1007/s00339-005-3424-9

2. Lapa, P. N., Ding, J., Pearson, J. E., Novosad, V., Jiang, J. S. & Hoffmann, A. Magnetization reversal in Py/Gd heterostructures. *Phys. Rev. B* **96**, 024418 (2017). https://doi.org/10.1103/PhysRevB.96.024418

3. Drovosekov, A.B., Savitsky, A.O., Kholin, D.I., Kreines, N.M., Proglyado, V.V., Makarova, M.V., Kravtsov, E.A. & Ustinov, V.V. Twisted magnetization states and inhomogeneous resonance modes in a Fe/Gd ferrimagnetic multilayer. *J. Magn. Magn. Mater.* **475**, 668 (2019). https://doi.org/10.1016/j.jmmm.2018.12.022

4. Becker, J., Tsukamoto, A., Kirilyuk, A., Maan, J. C., Rasing, Th., Christianen, P. C. M. & Kimel, A. V. Ultrafast Magnetism of a Ferrimagnet across the Spin-Flop Transition in High Magnetic Fields. *Phys. Rev. Lett.* **118**, 117203 (2017). https://doi.org/10.1103/PhysRevLett.118.117203
5. Mishra, R., Yu, J., Qiu, X., Motapothula, M., Venkatesan, T. & Yang, H. Anomalous Current-Induced Spin Torques in Ferrimagnets near Compensation. *Phys. Rev. Lett.* **118**, 167201 (2017). https://doi.org/10.1103/PhysRevLett.118.167201

6. Okuno, T., Kim, K.-J., Tono, T., Kim, S., Moriyama, T., Yoshikawa, H., Tsukamoto, A. & Ono, T. Temperature dependence of magnetoresistance in GdFeCo/Pt heterostructure. *Appl. Phys. Express* **9** 073001 (2016). http://doi.org/10.7567/APEX.9.073001

7. Harder, M., Gui, Y., Hu, C. M. Electrical detection of magnetization dynamics via spin rectification effects. *Physics Reports* **661**, 1 (2016). https://doi.org/10.1016/j.physrep.2016.10.002

8. Keller, S., Greser, J., Schweizer, M. R., Conca, A., Lauer, V., Dubs, C., Hillebrands, B. & Papaioannou, E. Th. Relative weight of the inverse spin-Hall and spin-rectification effects for metallic polycrystalline Py/Pt, epitaxial Fe/Pt, and insulating YIG/Pt bilayers: Angular dependent spin pumping measurements. *Phys. Rev. B* **96**, 024437 (2017). https://doi.org/10.1103/PhysRevB.96.024437

9. Khodadadi, B., Mohammadi, J. B., Mewes, C., Mewes, T., Manno, M., Leighton, C. & Miller, C. W. Enhanced spin pumping near a magnetic ordering transition. *Phys. Rev. B* **96**, 054436 (2017). https://doi.org/10.1103/PhysRevB.96.054436

10. Ranchal, R., Aroca, C., López, E. In-plane magnetotransport properties of Permalloy/gadolinium/Permalloy trilayers. *J. Appl. Phys.* **100**, 103903 (2006). https://doi.org/10.1063/1.2386931

11. Jungfleisch, M. B. et al. Thickness and power dependence of the spin-pumping effect in $Y_3Fe_5O_{12}$/Pt heterostructures measured by the inverse spin Hall effect. *Phys. Rev. B* **91**, 134407 (2015).

12. Iguchi, R. & Saitoh, E. Measurement of Spin Pumping Voltage Separated from Extrinsic Microwave Effects. *J. Phys. Soc. Jpn.* **86**, 011003 (2017). https://doi.org/10.7566/JPSJ.86.011003

13. Tsukahara, A., Ando, Y., Kitamura, Y., Emoto, H., Shikoh, E., Delmo, M. P., Shinjo, T. & Shiraishi, M. Self-induced inverse spin Hall effect in permalloy at room temperature. *Phys. Rev. B* **89**, 235317 (2014). https://doi.org/10.1103/PhysRevB.89.235317

14. Miao, B. F., Huang, S. Y., Qu, D. & Chien, C. L. Inverse Spin Hall Effect in a Ferromagnetic Metal. *Phys. Rev. Lett.* **111**, 066602 (2013). https://doi.org/10.1103/PhysRevLett.111.066602

15. Donahue, M. J. & Porter, D. G. OOMMF User's Guide Version 1.0 (National Institute of Standards and Technology, Gaithersburg, MD, 1999). The code is available at http://math.nist.gov/oommf.

16. Lapa, Pavel N., Ding, J., Pearson, John E., Novosad, V., Jiang, J. S. & Hoffmann A. Magnetization reversal in Py/Gd heterostructures. *Phys. Rev. B* **96**, 024418 (2017). https://doi.org/10.1103/PhysRevB.96.024418

17. McMichael, R. D. & Stiles, M. D. Magnetic normal modes of nanoelements. *J. Appl. Phys*. **97**, 10J901 (2005). https://doi.org/10.1063/1.1852191

18. Deng, J., Zhu, M., Luo, J., Zheng, H., Zheng, P., Feng, C. & Luo, J. Modulation of Magnonic Bands of Dipole-Exchange Spin Waves in Fishbone-like YIG Nanostrip Magnonic Crystal Waveguides. *J. Phys. D: Appl. Phys.* **53**, 315001 (2020). https://doi.org/10.1088/1361-6463/ab84aa
